# Tumor stromal type is associated with stromal PD-L1 expression and predicts outcomes in breast cancer

**DOI:** 10.1371/journal.pone.0223325

**Published:** 2019-10-04

**Authors:** Qinglian Zhai, Jiawen Fan, Qiulian Lin, Xia Liu, Jinting Li, Ruoxi Hong, Shusen Wang

**Affiliations:** 1 Sun Yat-sen University Cancer Center, State Key Laboratory of Oncology in South China, Collaborative Innovation Center for Cancer Medicine, Guangzhou, China; 2 Key Laboratory of Carcinogenesis and Translational Research (Ministry of Education/Beijing), Laboratory of Molecular Oncology, Peking University Cancer Hospital & Institute, Beijing, China; The University of Newcastle, AUSTRALIA

## Abstract

**Background/Aim:**

The aim of this study is to determine the relationship between stromal types, PD-L1 status and clinicopathological characteristics in patients with different molecular subtypes of breast cancer.

**Materials and methods:**

Protein expression levels of PD-L1 were determined by immunohistochemistry assay. Stromal type was classified based on the maturity of the tumor stroma.

**Results:**

Different subtypes of breast cancer had distinct stromal types. Tumors from patients with mature stroma had lower pathological N stage and AJCC stage, more frequent high p53 expression and positive stromal PD-L1 staining. Hormone receptor negative patients had higher frequency of positive stromal PD-L1 staining. Stromal PD-L1 status was also associated with different breast cancer subtypes and EGFR expression level. Importantly, our data revealed that stromal types and stromal PD-L1 status were independent prognostic factors.

**Conclusion:**

This study highlighted the importance of stromal types and stromal PD-L1 status in determining clinical outcomes in patients with breast cancer, and suggested that stromal type classification might be readily incorporated into routine clinical risk assessment following curative resection or optimal therapeutic design.

## Introduction

Breast cancer associated stroma is consisted of fibroblasts, myofibroblasts, leukocytes, endothelial cells, macrophages, adipocytes and extracellular matrix (ECM) [1]. Current clinical management guidelines for the determination of optimal treatment of breast cancer is mainly based on the characteristics of tumor (lymph node status, pathological stage, tumor size, grade, locoregional spread, and molecular features) [2]. However, the significance of tumor stroma is often overlooked, which contains valuable information for treatment choices. Andrew H. Beck and colleagues have revealed that the stromal compartment of breast tumors contains more prognostic information than the epithelial component [3]. Based on the principal stromal tissue component, Soomin Ahn and colleague classified tumor stroma into three dominant types, including collagen dominant, fibroblast dominant and lymphocyte dominant. They found that this classifier can stratify the prognostic outcome of breast cancer [2]. Other researchers proposed that stromal regions could be characterized according to the maturity of collagen in ECM. Depending upon the qualitative characteristics of the stromal collagen in the reactive tumor area, Ueno, et al [4] histologically classified stromal types into three categories: mature—when the stroma was composed of mature collagen fibers (fine and elongated fibers into multiple layers); intermediate—when keloid-like collagen was intermingled with mature fibers; and immature—consisting of a myxoid stroma in which no mature fibers were included. This study indicated that stromal response is critical for the tumor behavior and host immune reactions in rectal cancer and would be a promising tool for prognostic outcome prediction [4]. Breast cancer is a fibrotic cancer [5], the stromal types have not been extensively studied yet, especially the relationship between molecular subtypes (luminal A, luminal B, HER-2–enriched and triple negative breast cancer). Therefore, histologically characterizing the stromal types of different subtypes of breast cancer and analyzing their correlations may provide valuable information for clinical practice.

The host immune response has been reported to play a crucial role in breast cancer progression and response to therapy [6]. It is well recognized that the immune checkpoint pathways, such as those mediated by the programmed cell death protein 1 (PD-1) and programmed death ligand 1 (PD-L1), are significant for the antitumor responses [7, 8]. PD-L1 can be expressed by tumor cells as well as stromal cells, including the infiltrating T cells, macrophages, dendritic cells and B cells [9]. The protein level of tumor expressed PD-L1 has been demonstrated to be associated with high grade, hormone receptor–negative phenotypes [10], prognostic outcome [11], lymph node status [12] and immune cell infiltration [13]. However, there were few studies focusing on the relationship between stromal PD-L1 expression and stromal types and their correlation with clinicopathological characteristics.

The tumor microenvironment is well constructed and each component should have great influences on each other. It is reasonable to speculate that different molecular subtypes of breast cancer should have different stromal types and stromal PD-L1 expression and stromal types may associate with the expression patterns of PD-L1. In this perspective, we evaluated the stromal types of 160 breast cancers samples, which were stratified into different molecular subtypes. Additionally, the tumoral and stromal expressed PD-L1 was examined by immunohistochemistry. Interestingly, we found that stromal types were significantly associated with molecular subtypes of breast cancer, pathological N stage, American Joint Committee on Cancer (AJCC) stage, PR status, p53 level and stromal PD-L1 expression status. Meanwhile, stromal PD-L1 expression status was implicated with ER status, PR status, EGFR expression level and different molecular subtypes of breast cancer. Importantly, stromal types and stromal PD-L1 status were linked to the prognostic outcome. These morphological and molecular studies extended our knowledge on the microenvironment of breast cancer and provided meaningful data for the determination of optimal treatment of breast cancer, especially for immunotherapy decision.

## Materials and methods

### Patient characteristics and immunohistochemistry

Serial sections of tissue microarrays (TMAs) of 160 breast cancer specimens were obtained from Shanghai Outdo Biotech Co., Ltd. (SOBC), with the approval of the Institutional Review Board. Written informed consent was obtained from all patients prior to the study. The use of the clinical specimens for research purposes was approved by the Institutional Research Ethics Committee of Beijing Cancer Hospital and the Cancer Institute and Hospital (2019KT22).

All the patients enrolled here are therapeutic naïve. Follow-up data were available and the median follow-up time was 118 months. Clinical information including age, tumor grade, tumor location, pathological T stage, pathological N stage, AJCC stage, and survival data was evaluated by reviewing medical records. Overall survival (OS) was defined as the interval between the operation and death from breast cancer or the date of final follow-up [14]. Immunohistochemistry (IHC) analysis was performed and diagnosed by two pathologists blindly on the TMAs. Hematoxylin–eosin stained was performed by Shanghai Outdo Biotech Co., Ltd. (SOBC). The immunohistochemistry assay (IHC) was performed as previous described [15]. Tissue sections were incubated with rabbit anti–PD-L1 (1:100; Cell Signaling Technology, USA), overnight at 4°C. After washing, the tissue sections were treated with goat anti-mouse/rabbit IgG HRP-polymer (Agilent Dako, California, USA) for 30 min. 3, 3’-Diaminobenzidine was used as the chromogen. IHC scores were determined by combining the intensity of staining and the proportion of positively stained tumor cells. First, the intensity was graded as follows: 0, negative; 1, weak; 2, moderate; 3, strong. Second, the proportion of positive tumor cells was graded: 0, <5%; 1, 5–25%; 2, 26–50%; 3, 51–75%; 4,>75%. A final score was derived by multiplication of these two primary scores. Final scores of 0–3 were defined as ‘Negative’ (-); scores of 4–12 as ‘Positive’ (+). IHC staining of ER (1:200; Dako, Glostrup, Denmark), PR (1:200; Dako), HER2 (1:200; Dako), AR (androgen receptor, 1:100, Dako), EGFR (1:200, Dako), p53 (1:200, Dako), Ki-67 (1:200; Dako), CK-5/6 (1:100, Dako) and Fluorescence In Situ Hybridization (FISH) of HER2 were performed and evaluated by two pathologists by Shanghai Outdo Biotech Co., Ltd. (SOBC).

### Fluorescence In Situ Hybridization (FISH) of HER2

HER2 amplification was determined by FISH testing on a 4 μm formalin fixed paraffin-embedded tissue specimens using the FDA approved PathVysion HER2 DNA probe kit (Abbott Molecular, Des Plaines, IL, USA) according to the manufacturer’s instruction. HER2 FISH images were analyzed independently by two pathologists and HER2 amplification was defined by HER2-to-chromosome 17 centromere (CEP17) FISH ratio≥2.0 [16].

### Molecular subtypes classification

Molecular subtypes of breast cancer were surrogated by IHC markers and were defined as below [17,18]: Luminal A: ER (+), PR (+), HER2 (−), and Ki-67 ≤14%. Luminal B: ER (+), PR (+), HER2 (−), and Ki-67 >14%; ER (+), PR (+), HER2 (high or amplification). HER2-enriched: ER (−), PR (−), HER2 (+), and Ki-67 (high). Triple-negative: ER (−), PR (−), and HER2 (−). Basal like: triple-negative with CK5/6 high expression. AR+_ER/PR-: AR (+), ER (-), PR (-). AR+_TNBC: AR (+), ER (−), PR (−), and HER2 (−).

### Stromal types classification

The criteria of stromal types classification used in this study was described in the previous study [4]. Briefly, the fibrotic cancer stroma was classified into three types: mature, whose stroma was stratified into multiple layers by fine and elongated fibers and fibrocytes; intermediate, whose stroma was composed of broad bands of collagen with brightly eosinophilic hyalinization; immature, whose stroma consisted of randomly orientated keloid-like collagen bundles surrounded by myxoid stroma.

### Statistical analysis

Statistical analysis was carried out using IBM SPSS Statistics 20 or GraphPad Prism 8.0 for Windows. The two tailed Pearson χ^2^ test or Fisher’s exact test was used to correlate the stromal types or PD-L1 expression status with clinicopathological parameters. The survival curves were plotted by using Kaplan-Meier analysis and compared by log-rank test. Univariate Cox proportional hazards regression model was used to determine the hazard ratio (HR) between different stratified groups. To evaluate whether tumor PD-L1 expression, stromal PD-L1 expression and stromal types can serve as an independent prognostic factor in our cohort, age, pathological T stage, pathological N stage and pathological grade, which were prognostic associated factors, were taken into account as covariates for multivariate Cox regression analysis. Differences were considered significant when the p value was less than 0.05.

## Results

### Stromal types in breast cancer

**Fig 1:**
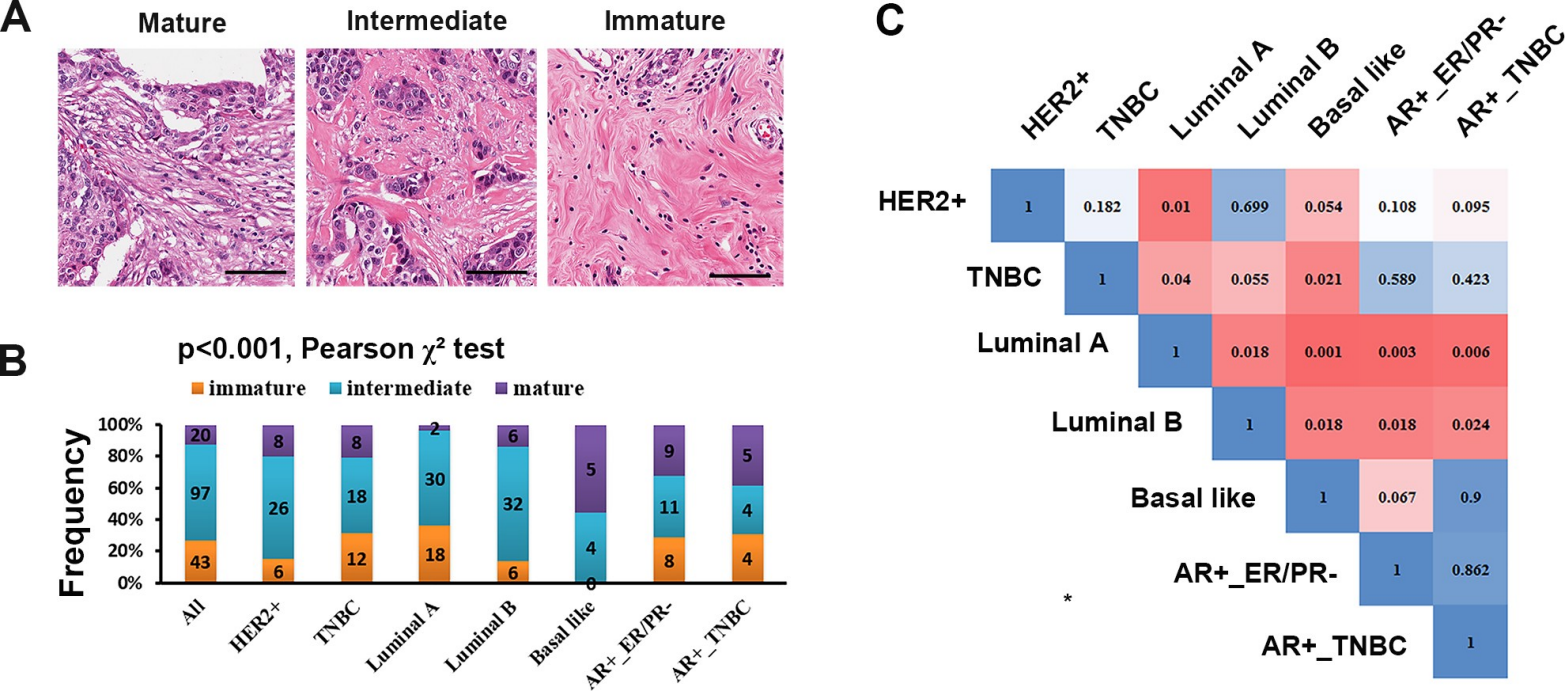
Fibrotic stromal types in different subtypes of breast cancer. (**A**) Maturation of breast cancer stroma. Scale bar: 50 μm. (**B**) Distributions of stromal types in different molecular subtypes of breast cancer. The number in the histogram bar indicated the number of cases. Multiple comparison among different molecular subtypes was performed by Pearson χ^2^ test. (**C)** Differences between each other of stromal types constitution among seven molecular subtypes indicated by p values. The blue to red gradient ramp indicates a p value from 1 to 0.001. The comparison between each molecular subtype was conducted by Pearson χ^2^ test and corrected by Bonferroni correction. Because of 21 comparisons between each other among seven molecular subtypes, differences were considered significant when the Bonferroni correction p value less than 0.0024 (0.05/21).

In our data set, 12.5% of patients had mature fibrotic cancer stroma whereas 60.6% had intermediate stroma and 26.9% had immature stroma (Fig 1A and 1B, Table 1). Different molecular subtypes had distinct stromal types (Pearson χ^2^ test, p<0.001, Fig 1B, Table 1), and the comparison between each molecular subtype by Pearson χ^2^ test and corrected by Bonferroni correction showed that the stromal types in Luminal A and Basal like were significantly different (Fig 1C, p = 0.001). Additionally, we observed that tumor stroma of HER2 (+) subtype had higher frequency of mature type, while lower frequency of immature type (p = 0.064). It was likely that tumors of progesterone receptor positive types (PR+) tended to have lower frequency of mature stroma (p = 0.014, Table 1). Tumors of Luminal A subtype showed lower frequency of mature stroma (p = 0.018, Table 1), however, tumors of Basal-like (p = 0.001, Table 1), AR+_ER/PR-(p = 0.004, Table 1), AR+_TNBC (p = 0.021, Table 1) subtypes had higher frequency of mature stroma. Additionally, tumors of Luminal B and Basal-like subtypes seemed to have lower frequency of immature stroma (p = 0.05 and p = 0.001, respectively. Table 1). Statistical analysis revealed that tumors with lower pathological N stage and AJCC stage accounted for higher frequency of mature stroma stromal types (p = 0.004 and p = 0.017, respectively. Table 1). Interestingly, breast cancer with mature stroma tended to have high p53 expression (p = 0.003, Table 1). However, stromal type was not correlated with the expression level of EGFR (p = 0.846, Table 1).

**Table 1:** Patient characteristics and the inter-relationship between clinicopathological characteristics and tumor stromal types in patients breast cancer (n = 160).

Feature		N (%)		Stromal types		
			Mature (%)	Intermediate (%)	Immature (%)	p value
Age	< = 50	82 (51.2)	9 (11.0)	53 (64.6)	20 (24.4)	0.564
	>50	78 (48.8)	11 (14.1)	44 (56.4)	23 (29.5)	
Tumor location	Right side	71 (44.4)	5 (7.0)	50 (70.4)	16 (22.5)	**0.046**
	Left side	89 (55.6)	15 (16.9)	47 (52.8)	27 (30.3)	
Grade	I	18 (11.2)	0 (0.0)	10 (55.6)	8 (44.4)	0.131
	II	136 (85.0)	19 (14.0)	83 (61.0)	34 (25.0)	
	III	6 (3.8)	1 (16.7)	4 (66.7)	1 (16.7)	
pT	pT1-2	36 (22.8)	2 (5.6)	24 (66.7)	10 (27.8)	0.279
	pT3-4	122 (77.2)	18 (14.8)	71 (58.2)	33 (27.0)	
pN	pN0-1	111 (71.2)	19 (17.1)	60 (54.1)	32 (28.8)	**0.004**
	pN2-3	45 (28.8)	1 (2.2)	35 (77.8)	9 (20.0)	
LNM	Yes	93 (59.6)	11 (11.8)	59 (63.4)	23 (24.7)	0.73
	No	63 (40.4)	9 (14.3)	36 (57.1)	18 (28.6)	
AJCC stage	1+2	107 (69.0)	18 (16.8)	58 (54.2)	31 (29.0)	**0.017**
	3	48 (31.0)	2 (4.2)	36 (75.0)	10 (20.8)	
HER2	Positive	40 (25.0)	8 (20.0)	26 (65.0)	6 (15.0)	0.064
	Negative	120 (75.0)	12 (10.0)	71 (59.2)	37 (30.8)	
ER	Positive	94 (58.8)	8 (8.5)	61 (64.9)	25 (26.6)	0.17
	Negative	66 (41.2)	12 (18.2)	36 (54.5)	18 (27.3)	
PR	Positive	78 (48.8)	4 (5.1)	53 (67.9)	21 (26.9)	**0.014**
	Negative	82 (51.2)	16 (19.5)	44 (53.7)	22 (26.8)	
AR	Positive	102 (63.7)	16 (15.7)	60 (58.8)	26 (25.5)	0.24
	Negative	58 (36.2)	4 (6.9)	37 (63.8)	17 (29.3)	
TNBC	Yes	38 (23.8)	8 (21.1)	18 (47.4)	12 (31.6)	0.105
	No	122 (76.2)	12 (9.8)	79 (64.8)	31 (25.4)	
Luminal A	Yes	50 (31.6)	2 (4.0)	30 (60.0)	18 (36.0)	**0.018**
	No	108 (68.4)	18 (16.7)	67 (62.0)	23 (21.3)	
Luminal B	Yes	44 (27.5)	6 (13.6)	32 (72.7)	6 (13.6)	**0.05**
	No	116 (72.5)	14 (12.1)	65 (56.0)	37 (31.9)	
Basal like	Yes	9 (5.6)	5 (55.6)	4 (44.4)	0 (0.0)	**0.001**
	No	151 (94.4)	15 (9.9)	93 (61.6)	43 (28.5)	
AR+_ER/PR-	Yes	28 (17.5)	9 (32.1)	11 (39.3)	8 (28.6)	**0.004**
	No	132 (82.5)	11 (8.3)	86 (65.2)	35 (26.5)	
AR+_TNBC	Yes	13 (8.1)	5 (38.5)	4 (30.8)	4 (30.8)	**0.021**
	No	147 (91.9)	15 (10.2)	93 (63.3)	39 (26.5)	
p53	High	36 (22.5)	11 (30.6)	17 (47.2)	8 (22.2)	**0.003**
	Low	124 (77.5)	9 (7.3)	80 (64.5)	35 (28.2)	
EGFR	High	40 (25.3)	6 (15.0)	23 (57.5)	11 (27.5)	0.846
	Low	118 (74.7)	14 (11.9)	73 (61.9)	31 (26.3)	
Tumor PD-L1	Positive	11 (7.4)	2 (18.2)	8 (72.7)	1 (9.1)	0.311
	Negative	138 (92.6)	18 (13.0)	81 (58.7)	39 (28.3)	
Stromal PD-L1	Positive	29 (19.5)	12 (41.4)	16 (55.2)	1 (3.4)	**<0.001**
	Negative	120 (80.5)	8 (6.7)	73 (60.8)	39 (32.5)	

### Correlation between stromal types and stromal PD-L1 expression status

**Fig 2:**
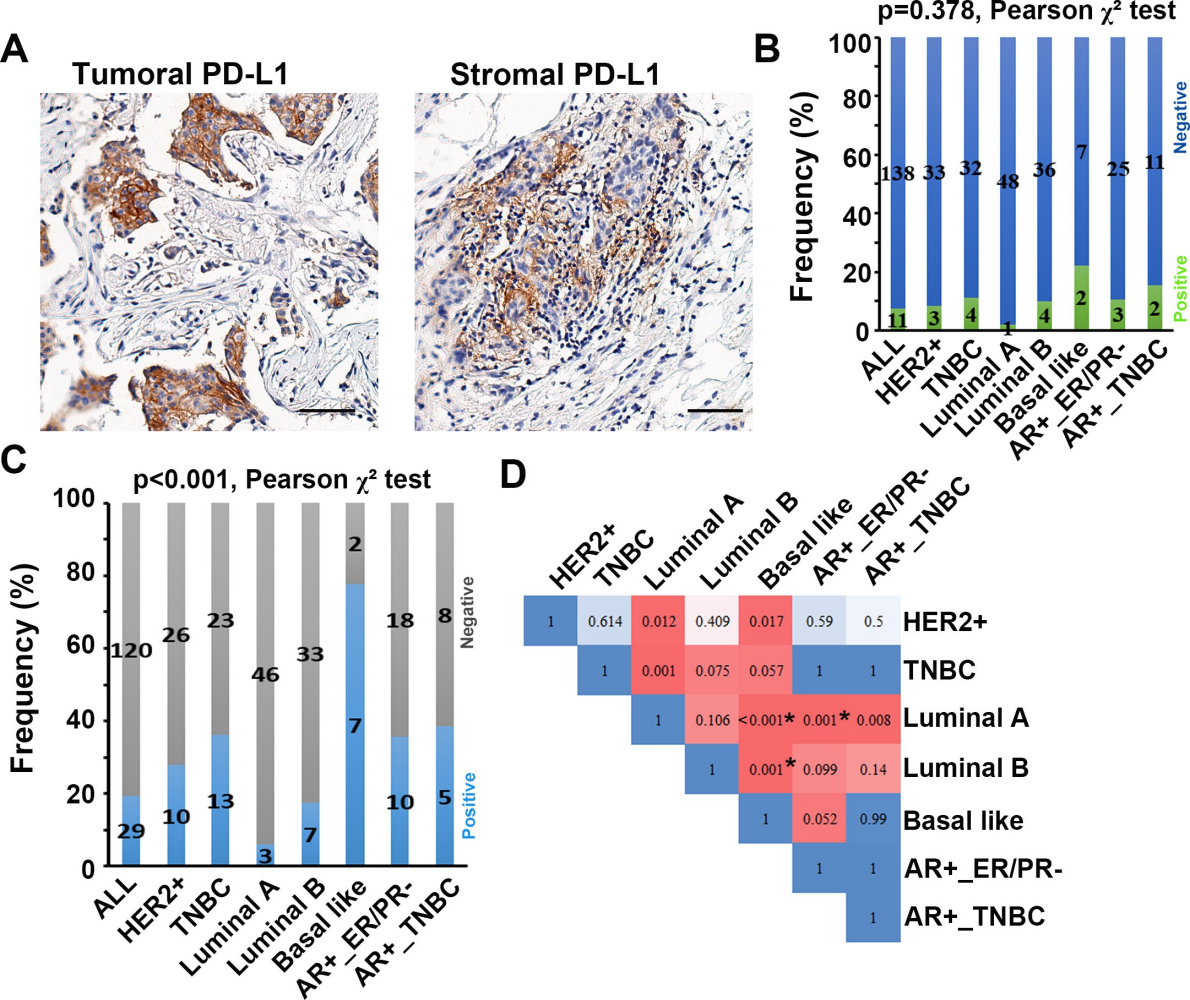
Tumoral and stromal expression of PD-L1. (**A**) Representative image of positive tumoral and stromal expression of PD-L1. Scale bar: 50 μm. (**B, C**) Distributions of tumoral(B) and stromal(C) PD-L1 expression status in different molecular subtypes of breast cancer. The number in the histogram bar indicated the number of cases. Multiple comparison among different molecular subtypes was performed by Pearson χ^2^ test. (**D**) Differences between each other of stromal PD-L1 expression status among seven molecular subtypes indicated by p values. The comparison between each molecular subtype was conducted by Pearson χ^2^ test and corrected by Bonferroni correction. Because of 21 comparisons between each other among seven molecular subtypes, differences were considered significant when the Bonferroni correction p value less than 0.0024 (0.05/21).

The percentage of patients with positive PD-L1 expression were relative low, which was 7.4% for tumor cells and 19.5% for stromal cells (Fig 2). Intriguingly, the stromal type was significantly associated with stromal PD-L1 status, with mature fibrotic cancer stroma having the highest rate of positive stromal PD-L1 expression (p<0.001, Table 2). However, there was no statistically significant difference between stromal types and tumoral PD-L1 status (p = 0.311, Table 2). To evaluate whether tumoral and stromal PD-L1 expression status were significantly different in different molecular subtypes of breast cancer, we performed Pearson χ^2^ test for multiple comparisons. The result showed that tumoral PD-L1 expression status was not significantly different among different molecular subtypes (p = 0.378, Fig 2B), while stromal PD-L1 expression status was significantly different among different molecular subtypes (p<0.001, Fig 2C). To further distinguish the difference between each molecular subtype, Pearson χ^2^ test and Bonferroni correction was conducted. It revealed that stromal PD-L1 expression status was significantly different between Luminal A and Basal like subtypes (p<0.001), Luminal A and AR+_ER/PR- subtypes (p = 0.001), Luminal B and Basal like (p = 0.001). Taken together, stromal subtypes might have a great influence on the PD-L1 expression in tumor microenvironmental cells.

**Table 2:** The inter-relationship between clinicopathological characteristics and PD-L1 expression status in patients breast cancer (n = 160).

Feature		N (%)	Tumor PD-L1			Stromal PD-L1		
			Positive (%)	Negative (%)	p value	Positive (%)	Negative (%)	p value
Age	< = 50	77 (51.7)	3 (3.9)	74 (96.1)	0.092	13 (16.9)	64 (83.1)	0.411
	>50	72 (48.3)	8 (11.1)	64 (88.9)		16 (22.2)	56 (77.8)	
Tumor location	Right side	65 (43.6)	5 (7.7)	60 (92.3)	0.899	14 (21.5)	51 (78.5)	0.574
	Left side	84 (56.4)	6 (7.1)	78 (92.9)		15 (17.9)	69 (82.1)	
Grade	I	16 (10.7)	0 (0.0)	16 (100.0)	0.192	2 (12.5)	14 (87.5)	0.718
	II	129 (86.6)	11 (8.5)	118 (91.5)		26(20.2)	103 (79.8)	
	III	4 (2.7)	0 (0.0)	4 (100.0)		1(25.0)	3(75.0)	
pT	pT1-2	32 (21.8)	3 (9.4)	29 (90.6)	0.646	6 (18.8)	26 (81.2)	0.875
	pT3-4	115 (78.2)	8 (7.0)	107 (93.0)		23 (20.0)	92 (80.0)	
pN	pN0-1	103 (70.5)	8 (7.8)	95 (92.2)	0.869	23 (22.3)	80 (77.7)	0.248
	pN2-3	43 (29.5)	3 (7.0)	40 (93.0)		6 (14.0)	37 (86.0)	
LNM	Yes	90 (61.6)	4 (4.4)	86 (95.6)	0.073	14 (15.6)	76 (84.4)	0.098
	No	56 (38.4)	7 (12.5)	49 (87.5)		15 (26.8)	41 (73.2)	
AJCC stage	1+2	99 (68.3)	8 (8.1)	91 (91.9)	0.741	23 (23.2)	76 (76.8)	0.153
	3	46 (31.7)	3 (6.5)	43 (93.5)		6 (13.0)	40 (87.0)	
HER2	Positive	36 (24.2)	3 (8.3)	33 (91.7)	0.802	10 (27.8)	26 (72.2)	0.148
	Negative	113 (75.8)	8 (7.1)	105 (92.9)		19 (16.8)	94 (83.2)	
ER	Positive	89 (59.7)	5 (5.6)	84 (94.4)	0.316	10 (11.2)	79 (88.8)	**0.002**
	Negative	60 (40.3)	6 (10.0)	54 (90.0)		19 (31.7)	41 (68.3)	
PR	Positive	71 (47.7)	2 (2.8)	69 (97.2)	**0.042**	6 (8.5)	65 (91.5)	**0.001**
	Negative	78 (52.3)	9 (11.5)	69 (88.5)		23 (19.5)	55 (70.5)	
AR	Positive	95 (63.8)	6 (6.3)	89 (93.7)	0.509	16 (16.8)	79 (83.2)	0.284
	Negative	54 (36.2)	5 (9.3)	49 (90.7)		13 (24.1)	41 (75.9)	
TNBC	Yes	36 (24.2)	4 (11.1)	32 (88.9)	0.326	13 (36.1)	23 (63.9)	**0.004**
	No	113 (75.8)	7 (6.2)	106 (93.8)		16 (14.2)	97 (85.8)	
Luminal A	Yes	49 (33.1)	1 (2.0)	48 (98.0)	0.079	3 (6.1)	46 (93.9)	**0.004**
	No	99 (66.9)	10 (10.1)	89 (89.9)		26 (26.3)	73 (73.7)	
Luminal B	Yes	40 (26.8)	4 (10.0)	36 (90.0)	0.459	7 (17.5)	33 (82.5)	0.714
	No	109 (73.2)	7 (6.4)	102 (93.6)		22 (20.2)	87 (79.8)	
Basal like	Yes	9 (6.0)	2 (22.2)	7 (77.8)	0.079	7 (77.8)	2 (22.2)	**<0.001**
	No	140 (94.0)	9 (6.4)	131 (93.6)		22 (15.7)	118 (84.3)	
AR+_ER/PR-	Yes	28 (18.8)	3 (10.7)	25 (89.3)	0.454	10 (35.7)	18 (64.3)	**0.016**
	No	121 (81.2)	8 (6.6)	113 (93.4)		19 (15.7)	102(84.3)	
AR+_TNBC	Yes	13 (8.7)	2 (15.4)	11 (84.6)	0.248	5 (38.5)	8 (61.5)	0.07
	No	136 (91.3)	9 (6.6)	127 (93.4)		24 (17.6)	112 (82.4)	
p53	High	33 (22.1)	3 (9.1)	30 (90.9)	0.671	9 (27.3)	24 (72.7)	0.199
	Low	116 (77.9)	8 (6.9)	108 (93.1)		20 (17.2)	96 (82.8)	
EGFR	High	37 (25.2)	5 (13.5)	32 (86.5)	0.107	14 (37.8)	23 (62.2)	**0.001**
	Low	110 (74.8)	6 (5.5)	104 (94.5)		15 (13.6)	95 (86.4)	
Stromal types	mature	20 (13.4)	2 (10.0)	18 (90.0)	0.311	12 (60.0)	8 (40.0)	**<0.001**
	intermediate	89 (59.7)	8 (9.0)	81 (91.0)		16 (18.0)	73 (82.0)	
	immature	40 (26.8)	1 (2.5)	39 (97.5)		1 (2.5)	39 (97.5)	

### Stromal PD-L1 status correlated with clinicopathological characteristics

Statistical analysis revealed that tumor PD-L1 status only negatively correlated with PR status (p = 0.042), while it was not significantly associated with any other clinicopathological features. However, stromal PD-L1 status was significantly associated with many clinicopathological characteristics (Table 2), representing that higher frequency of positive stromal PD-L1 in hormone receptor negative subtypes (p = 0.002 for ER- and p = 0.001 for PR-), TNBC subtype (P = 0.004), Basal like subtype (P<0.001), AR+_ER/PR- subtype (P = 0.016) and lower frequency of positive stromal PD-L1 in Luminal A subtype (p = 0.004). Intriguingly, high EGFR expression was correlated with high frequency of positive stromal PD-L1 staining (p = 0.001). These findings suggested that stromal PD-L1 was significantly affected by host tumor characteristics and might have important clinical implications.

### Stromal types and stromal PD-L1 status predicted prognostic outcome

**Fig 3:**
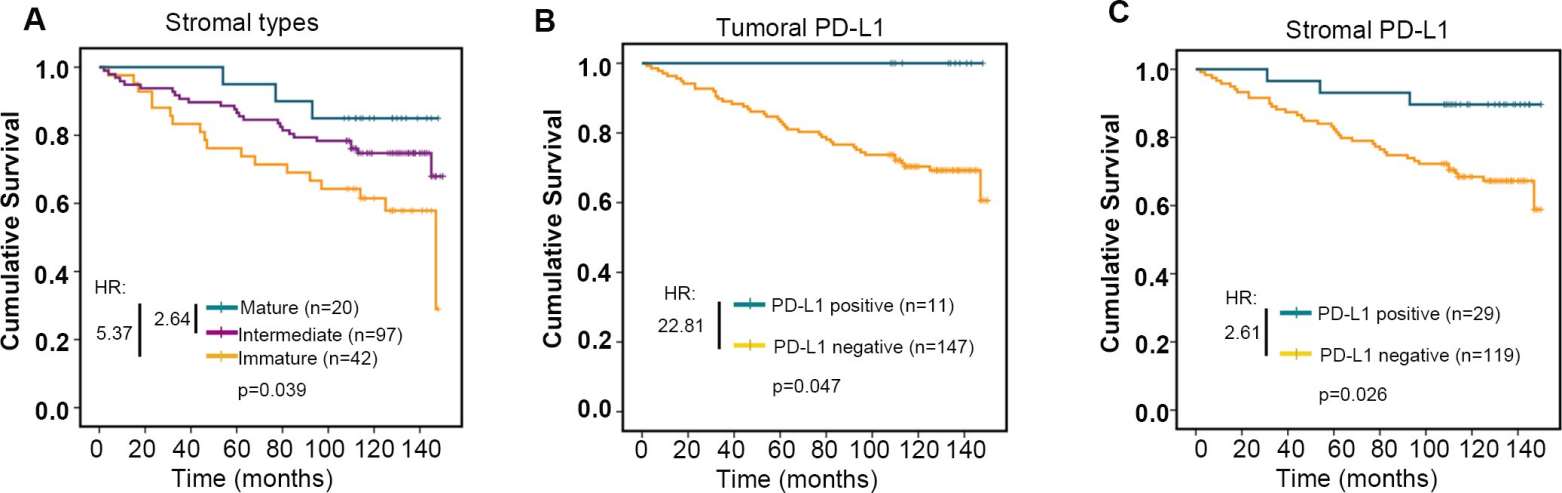
Prognostic value of stromal types and PD-L1 expression status. Kaplan-Meier survival analysis of patients with breast cancer stratified by stromal types (**A**), tumoral PD-L1 expression status (**B**) and stromal PD-L1 expression status (**C**). Log-rank test was used to evaluate differences between/among different groups. Univariate Cox proportional hazards regression model was used to determine the hazard ratio (HR) between different stratified groups.

Notably, stromal types were significantly associated with overall survival of breast cancer patients, whose tumor with mature stroma had the best overall survival, and with immature stroma had the worst survival (p = 0.039, Kaplan–Meier survival analysis and log-rank test, HR: mature stroma vs immature stroma was 5.37; intermediate stroma vs immature stroma was 2.64. Fig 3A). Kaplan–Meier survival analysis also revealed that patients with tumoral or stromal PD-L1 expression were linked to better survival outcome (p = 0.047 and p = 0.026, respectively, Kaplan–Meier survival analysis and log-rank test. HR was 22.81 and 2.61, respectively. Fig 3B and 3C). Interestingly, multivariate Cox regression survival analysis adjusting for stromal types, age, pathological T stage, pathological N stage and pathological grade consistently reported strong correlation between stromal types and overall survival (p = 0.012, HR = 0.441, 95% CI 0.234 to 0.832 for mature vs immature, and p = 0.085, HR = 0.333, %95 CI 0.095 to 1.164 for intermediate vs immature. Table 3), indicating that stromal type was an independent prognostic factor for outcome in breast cancer. Similarly, multivariate Cox regression survival analysis adjusting for stromal PD-L1 status, age, pathological T stage, pathological N stage and pathological grade also revealed that stromal PD-L1 status was an independent prognostic factor for outcome of breast cancer (p = 0.033, HR = 0.278, %95 CI 0.085 to 0.902 for positive vs negative, Table 4). However, when taking all these variables into consideration, multivariate Cox regression survival analysis failed to report that stromal types and stromal PD-L1 status predicted prognosis independently (Table 5).

**Table 3:** Multivariate Cox analysis on stromal types and other prognostic factors.

Clinical features	p value	HR	95% CI	
			Up	Down
Stromal type	**0.022**			
Stromal type (Mature vs Immature)	**0.012**	0.441	0.234	0.832
Stromal type (Intermediate vs Immature)	0.085	0.333	0.095	1.164
Age	**0.035**	1.938	1.049	3.579
Grade (I vs II+III)	0.758	0.857	0.323	2.279
Pathological T stage (T1+T2 vs T3+T4)	0.799	1.105	0.512	2.382
Pathological N stage (N0+N1 vs N2+N3)	**0.003**	0.378	0.198	0.723

**Table 4:** Multivariate Cox analysis on stromal PD-L1 and other prognostic factors.

Clinical features	p value	HR	95% CI	
			Up	Down
Age	**0.021**	2.129	1.120	4.045
Grade (I vs II+III)	0.343	0.620	0.231	1.666
Pathological T stage (T1+T2 vs T3+T4)	0.505	1.335	0.571	3.122
Pathological N stage (N0+N1 vs N2+N3)	**0.017**	0.457	0.240	0.869
stromal PD-L1	**0.033**	0.278	0.085	0.902

**Table 5:** Multivariate Cox analysis on stromal types, stromal PD-L1 and other prognostic factors.

Clinical features	p value	HR	95% CI	
			Up	Down
Age	**0.035**	1.984	1.051	3.748
Grade (I vs II+III)	0.551	0.734	0.266	2.026
Pathological T stage (T1+T2 vs T3+T4)	0.439	1.412	0.589	3.384
Pathological N stage (N0+N1 vs N2+N3)	**0.014**	0.427	0.217	0.840
stromal PD-L1	0.114	0.363	0.103	1.273
Stromal types	0.053			
Stromal type (Mature vs Immature)	**0.017**	0.445	0.229	0.864
Stromal type (Intermediate vs Immature)	0.280	0.477	0.125	1.827

## Discussion

Breast cancer is a fibrotic/desmoplastic stroma cancer [5, 19], and tumor stroma percentage (TSP) or tumor-stroma ratio (TSR) has been recently reported to have prognostic value in patients with triple negative [20] and node-negative breast cancer [21]. However, the relationship between fibrotic stromal types and clinicopathological features has not been extensive studied yet. Here, we investigated the stromal types and molecular subtypes of 160 breast cancer and explored the relationship with many important determinants of outcome such as age, grade, pathological T stage, N stage, lymph node metastasis, AJCC stage, overall survival status, tumoral and stromal PD-L1 status. Interestingly, stromal types were distinct among different molecular subtypes. Mature stromal type was significantly correlated with lower pathological N stage and AJCC stage and better prognostic outcome. Moreover, we also showed that positive stromal PD-L1 staining was enriched in patients with mature stromal type. These findings revealed that stromal type may not only be a candidate parameter for prognostication, but might also lead to the subsequent development of immuno-therapeutic strategies.

Apart from the tumor cell itself, the knowledge of the complex microenvironment will provide full understanding of the behaviors of tumor. Compelling evidence reveals that tumor stroma, one component of tumor microenvironment, remarkably affects tumor growth and progression [22, 23]. It is well studied that stroma promotes tumor proliferation and dissemination by multiple mechanisms, including remodeling extracellular matrix, recruiting of inflammatory cells, rewiring stromal regulatory pathways [24–27]. Previous studies have reported that tumor stroma was implicated with prognostic outcome in patients with colorectal [28, 29] and esophageal cancers [30]. Additionally, the percentage of tumor stroma has been recently reported to have prognostic value in patients with triple negative [20] and node-negative breast cancer [21]. However, in the basis of stromal maturity, there was just one study that reported that immature stroma of breast cancer was correlated with higher grade and positive nodes [31] Our study here showed that mature stroma of breast cancer was significantly associated with lower pathological N stage and AJCC stage, however, it did not come to statistical significance with grade and lymph node metastasis. Importantly, our results also provided evidence that different molecular subtypes of breast cancer have distinct stromal types. ER positive and PR positive tumor tended to have lower percentage of mature stroma, while the HER2 enriched subtype of tumor tended to contain a higher percentage of mature stroma. Additionally, the tumor microenvironment of Luminal A tumors consisted of less mature stroma than the other subtypes, while the tumor microenvironment of Luminal B tumors consisted of less immature stroma than the other subtypes. The percentage of mature stroma was significantly higher in basal like, AR positive-ER/PR negative, and AR positive-triple negative breast cancer. The distinct relationship between stromal types and molecular subtypes of breast cancer suggests that the behaviors of different molecular subtypes may be affected by different stromal types and stromal types should be taken into consideration when making optimal treatment choices, which is worthy and urgent need for further investigations.

Intriguingly, the percentage of tumoral p53 high expression was significantly higher in those surrounded with mature stroma. This phenomenon was consistent with previous studies which have revealed that p53 can inhibit collagen expression in fibroblast [32, 33]. Additionally, Sonja M. Wörmann et al also demonstrated that loss of p53 function in pancreatic tumor activated Shp2-JAK2–STAT3 signaling, which promoted desmoplasia [34]. The cell autonomous roles of the p53 protein have been comprehensively studied, while accumulating evidence suggests that p53 has a non-cell autonomous tumor suppressing role in the tumor stroma by regulating the expression of various secreted proteins [35]. Recently, Rong Fu et al revealed that ZEB1/p53 signaling axis in stromal fibroblasts could promote mammary epithelial tumors via enhancing FGF2/7, VEGF and IL-6 expression and secretion [36]. However, whether stromal elements interacting with tumor cells will influence the expression of p53 in tumor cells is still unclear, which is worthy to be in-depth studied.

The relationships between the gross pathological characteristics, tumor stroma and tumor microenvironment are extremely complex, however, the stromal types remained independently and strongly associated with overall survival of patients with breast cancer. These results confirmed the crucial role of tumor stroma in determining oncological outcome. Given that the determination of tumor stromal types is a relatively simple and quick procedure in risk assessment of breast cancer, it is promising that stromal maturity classification could serve as a tool for the routine pathological examination, which can be done on routine H&E sections without the necessity for further staining.

Our work also demonstrated that different molecular subtypes had different tumoral and stromal PD-L1 expression. TNBC and basal like subtypes tend to have a higher percentage of positive PD-L1 expression, while Luminal A subtype had the lowest positive staining of PD-L1, this phenomenon was consistent with the previous study [37] and suggesting further categorization of TNBC in subgroups according to the PD-L1 status will benefit immune checkpoint blockade.

In the present study, the tumor cells and stromal cells had low frequency of PD-L1 positive staining, however, the percentage of positive stromal PD-L1 staining was relatively higher than tumoral PD-L1 staining, which was consistent with the previous study in DCIS [38]. Recently, a meta-analysis including initial 4184 entries, 38 retrospective studies of breast cancer revealed that the overall pooled PD-L1 protein positivity rate was 24% (95% CI 15–64%) in tumor cells and 33% (95% CI 14–56%) in immune cells [37]. The heterogeneity in PD-L1 expression may be due to the study population, antibody used and positivity threshold, which is a major challenge that needs to be overcome before PD-L1 testing can be standardized and used in daily clinical practice. Additionally, the relatively low frequency of positive PD-L1 staining in breast cancer may be due to the limited detection technology. Heng-Huan Lee et al recently demonstrated that removal of N-linked glycosylation of PD-L1 could enhance the recognition of PD-L1 antibodies, and deglycosylated PD-L1 is a more reliable biomarker to guide immunotherapy [39]. On the other hand, the prognostic implications of PD-L1 in a large number of studies investigating breast cancer have had conflicting results. Here, we showed that positive stromal PD-L1 expression was correlated with better prognostic OS, which was consistent with the recent meta-analysis, while the correlation of tumoral PD-L1 expression status and OS was on the contrary[37]. This discrepancy may be due to the heterogeneous populations, and robust analysis and clinical validity test should be performed in a larger sample size. Meanwhile, we also found a significant correlation between stromal types and stromal PD-L1 status that positive stromal PD-L1 was strongly enriched in patients with mature stroma. Stroma types and stromal PD-L1 status were reported as independent prognostic factors when multivariate Cox regression survival analysis adjusted by age, pathological T stage, pathological N stage and pathological grade. However, when taken all these variates into consideration, stromal types or stromal PD-L1 status failed to predict prognosis independently, which indicating that stromal types had a direct effect on the expression of stromal PD-L1. These observations provided important insights for understanding immune-based treatment response, especially for the strategies of inhibitors of PD-L1 in breast cancer, and guiding the design and analysis of relevant clinical trials. Our finding also highlighted the importance of stromal types on immunological response in patients with breast cancer. Implementing this simple and reproducible parameter in routine pathological examination may help optimizing patient stratification for immune-based therapeutic strategies.

The stroma-rich tumor may hinder the penetration of monoclonal antibodies (mAbs) into the cells, and that leads to failure of the conventional cell-targeting immunoconjugate strategy. Our observation revealed that a subset of HER+ or PD-L1 positive patients, which may be benefit from anti-HER2 or anti-PD-L1 monoclonal antibodies therapy, possessed immature stroma. These patients may have no response for immunoconjugate strategy. To overcome this drawback, Masahiro Yasunaga et al designed a strategy that conjugated SN-38, a topoisomerase I inhibitor, to a mAb to collagen 4, a plentiful component of the tumor stroma via ester-bond, which was effective to stroma-rich pancreatic cancer[40]. Toward Hypervascular stroma-poor tumor, the authors generated an anti-CD20 mAb-PEG-SN-38 via carbamate-bond as conventional immunoconjugate, which promoted SN-38 to be released by a carboxylesterase inside of the tumor cell following the internalization, showed strong anti-tumor activity [40]. Another promising example is that Samaresh Sau et al conjugated PD-L1 antibody to Doxorubicin (Dox) through a hydrazone linker containing a polyethylene glycol (PEG) spacer. Dox was used to disrupt the tumor extracellular environment so that PD-L1 antibody can penetrate the tumor core. PD-L1-Dox demonstrates significant antitumor activity in a breast cancer spheroid model [41]. These studies raise the possibility that targeting tumor stroma via immunoconjugate strategy may be a promising choice for increasing treatment efficacy.

In conclusion, our study demonstrates the importance of stromal types and stromal PD-L1 status in determining clinical outcomes in patients with breast cancer. Due to its simple and quick assessment procure, stromal type classification may be readily incorporated into routine clinical risk stratification following curative resection or optimal therapeutic design. These findings also highlighted that stromal types should be taken into consideration in the determinant of different therapeutic strategies for different molecular subtypes, especially for immune-based therapy.
